# Feasibility Randomized Trial for an Intensive Memory-Focused Training Program for School-Aged Children with Acquired Brain Injury

**DOI:** 10.3390/brainsci10070430

**Published:** 2020-07-07

**Authors:** Monica Recla, Erika Molteni, Valentina Manfredi, Filippo Arrigoni, Andrea Nordio, Susanna Galbiati, Valentina Pastore, Marc Modat, Sandra Strazzer

**Affiliations:** 1 Neurophysiatric Department, Neuropsychological and Cognitive-behavioral Service, Scientific Institute, I.R.C.C.S. Eugenio Medea, 23842 Bosisio Parini, Italy; valentina.manfredi@lanostrafamiglia.it (V.M.); susanna.galbiati@lanostrafamiglia.it (S.G.); valentina.pastore@lanostrafamiglia.it (V.P.); 2 School of Biomedical Engineering & Imaging Sciences, and Centre for Medical Engineering, King’s College, London SE1 7EU, UK; erika.molteni@kcl.ac.uk (E.M.); marc.modat@kcl.ac.uk (M.M.); 3 Neuroimaging Lab, Scientific Institute, I.R.C.C.S. Eugenio Medea, 23842 Bosisio Parini, Italy; filippo.arrigoni@lanostrafamiglia.it (F.A.); andrea.nordio@lanostrafamiglia.it (A.N.); 4 Neurophysiatric Department, Scientific Institute, I.R.C.C.S. Eugenio Medea, 23842 Bosisio Parini, Italy; sandra.strazzer@lanostrafamiglia.it

**Keywords:** pediatric brain injury, child, memory, rehabilitation, immediate and delayed recall, left inferior frontal gyrus

## Abstract

(1) Background: Memory deficits are common sequelae of pediatric Acquired Brain Injury (ABI). Only methods for non-focused cognitive remediation are available to the pediatric field. The aims of this feasibility trial are the description, implementation, and test of an intensive program specific to the training and re-adaptation of memory function in children, called Intensive Memory-Focused Training Program (IM-FTP); (2) Methods: Eleven children and adolescents with ABI (mean age at injury = 12.2 years, brain tumor survivors excluded) were clinically assessed and rehabilitated over 1-month through IM-FTP, including physio-kinesis/occupational, speech, and neuropsychology treatments. Each patient received a psychometric evaluation and a brain functional MRI at enrollment and at discharge. Ten pediatric controls with ABI (mean age at injury = 13.8 years) were clinically assessed, and rehabilitated through a standard program; (3) Results: After treatment, both groups had marked improvement in both immediate and delayed recall. IM-FTP was associated with better learning of semantically related and unrelated words, and larger improvement in immediate recall in prose memory. Imaging showed functional modification in the left frontal inferior cortex; (4) Conclusions: We described an age-independent reproducible multidisciplinary memory-focused rehabilitation protocol, which can be adapted to single patients while preserving inter-subject comparability, and is applicable up to a few months after injury. IM-FTP will now be employed in a powered clinical trial.

## 1. Introduction

Pediatric acquired brain injury (ABI) arises as a result of trauma, infection, vascular catastrophe or neoplastic formation. The most common sequelae include attention and memory deficits, and decreased speed of information processing. Memory-related deficits include anterograde, retrograde, and implicit memory difficulties. As attention and memory functions reach full maturation during adolescence, brain injury during childhood not only disrupts existing established functions, but is also detrimental to further development [1]. The effectiveness of cognitive rehabilitation of adults surviving traumatic ABI has been demonstrated in several studies [2,3,4], while the pediatric field lags behind in validated methods for cognitive remediation. This is due to the huge methodological efforts needed to compensate for the developmental variability at different ages, to cope with language specificities, and to integrate the training activities with academic requirements.

One of the first programs specifically designed for children is the Amsterdam Memory and Attention Training for children (AMAT-c) [5,6]; this 18-weeks training plan differentiates between three levels of attention (sustained attention, selective attention and mental tracking), and three aspects of memory function (the sensory register, working memory and long-term memory), all integrated into a hierarchical model of intervention [7]. Two AMAT-c program versions are available, depending on the patient’s age (9–12 years; >13 years). The Swedish and Dutch versions of AMAT-c have been tested in pediatric cohorts with traumatic ABI [8,9,10]. In a randomized controlled study, the program was delivered to 38 children with ABI; improved complex attention and memory were observed as an immediate effect of the training program [7]. Improvement in tasks involving sustained and selective attention, coding, auditory verbal learning and verbal working memory, and freedom of distractibility, was maintained six months after completion of the cognitive training; conversely, no short-/long-term effects were demonstrated on measures of reaction time [9]. The unbalanced presence of cancer survivors in the groups, nevertheless, partially limited the interpretability of results. The English version of AMAT-c was initially tested on 3 cases [11] and later on 10 patients [12], with analogous findings. Both of these studies, however, lacked a comparative training program. In addition to AMAT-c, other pediatric programs for cognitive training have been published and tested, including computerized home-based tools; however, these have only been validated in children with specific ABI etiologies, such as cancer survivors [13,14].

Since the introduction of AMAT-c, the understanding of specific memory functions has been refined, with recent research showing that major repercussions after traumatic brain injury (TBI) involve verbal and visuo-spatial memory [15] and working memory [16]. Encouraging results have also been reported on the recovery of immediate visual memory in severe patients [17]. Notwithstanding, no validated training program has focused specifically on memory functions in pediatrics so far.

Memory has wide neurofunctional underpinnings, mainly involving the frontal and temporal lobes, and the basal ganglia [18]. Although neuropsychological studies support the effectiveness of memory training after TBI, the demonstration of neurofunctional modifications induced by specific treatments is much harder and largely unexplored in this field, due to the heterogeneous nature of each individual’s brain damage [19]. Nevertheless neuroimaging is indispensable for the investigation of the neuronal representations engaged during training and the network re-organization after training, as it permits distinguishing between *restorative* and *compensatory* effects [20]. Literature proved that meaningful task-specific rehabilitation, when combined with environmental enrichment, is capable of augmenting intrinsic neural plasticity within non-injured functionally connected brain regions, as well as of promoting functional outcome [21,22]. Of note, although repetition plays a major role in inducing and maintaining brain changes [23], repetitive activity alone may be insufficient to the induction of representational plasticity in the brain cortex, especially when the task is easy or re-learning is not needed [24] and/or when the task is neither purposeful, nor oriented to a goal [21].

To date, no pediatric tool for memory-focused intervention is available; children specifically sustaining a memory impairment after ABI are treated either through non focused pediatric programs such as AMAT-c or through adaptations of methods validated on adults. Our hypothesis is that an intensive and holistic rehabilitation program, focused on the training and re-adaptation of memory, could improve outcome.

The aims of this feasibility trial are: (i) the implementation of an intensive rehabilitation program, focused on the training and re-adaptation of memory, called Intensive Memory-Focused Training Program (IM-FTP); (ii) the assessment of the program efficacy in a group of patients of school-age with non-tumor ABI, through both psychometric tests and neuroimaging, and by comparison with a control group receiving a non-focused intervention; (iii) the assessment of the program feasibility in an in-patient rehabilitation setting, and during the post-acute stage of disease; (iv) the detailed description of the intervention and of its mode of employment, to favor replicability and dissemination among therapists working with pediatric patients with ABI.

To the authors’ knowledge, this is the first study employing functional neuroimaging for the assessment of memory intervention efficacy in pediatric ABI. Based on present state-of-the art knowledge of the neural correlates of memory [18], and on the specific memory functions trained by IM-FTP, we expect frontal reorganization following training.

## 2. Materials and Methods

### 2.1. Participants

A study group of 167 consecutive inpatients was enrolled in the period between 2013 and 2018. The participants were children and adolescents with acquired brain injury (ABI) referred to the Scientific Institute “I.R.C.C.S. Eugenio Medea” for clinical and functional assessment and for rehabilitation. Of these, 21 participants met the following inclusion criteria: (i) age at first assessment between 6 and 18 years; (ii) time between injury and first assessment <3 months; (iii) documented evidence of a severe ABI of traumatic or non-traumatic (i.e., anoxic, vascular or infectious) etiology, as indicated by a Glasgow Coma Scale (GCS, [25]), score ≤8 at injury or onset; (iv) presence of severe memory impairment, as assessed at first evaluation; (v) sufficient attentive skills for attending a simple task and understanding simple commands/directions, and sufficient verbal comprehension for executing simple procedures/exercises, based on *Levels of Cognitive Functioning Assessment Scale* (LCF) and the *Wechsler Intelligence Scale for Children-III* (WISC-III) or the *Wechsler Adult Intelligence Scale-Revised* (WAIS-R), according to the patients’ age; (vi) absence of congenital pathology or disability previous to the injury; (vii) medical records sufficiently detailed to determine the injury severity and neurological findings; (viii) absence of severe motor or sensitive deficits.

After obtaining parental informed consent, the selected patients were assigned to either the experimental or control group through electronic randomization (R script), while balancing the groups for gender, pre-treatment variables such as age at time of injury, and injury-to-treatment time. Eleven patients entered the experimental group and took part in the Intensive Memory-Focused Training Program (IM-FTP), focused on the re-adaptation of memory function. Ten patients entered the standard rehabilitation intervention, non-specifically addressing the recovery of the higher cognitive functions (motor, attention, memory and executive functions).

Seven participants, with no history of brain injury and age-matched with the experimental group, were recruited in the healthy group to undergo functional magnetic neuroimaging and provide a reference for neurofunctional activation.

The study [NCT04206475, registered at https://clinicaltrials.gov] was approved by the “I.R.C.C.S. Eugenio Medea” Ethical Committee [Id. No.08/19_Oss], in compliance with the Declaration of Helsinki. The feasibility trial ended when at least 10 patients were recruited in each arm of the study.

### 2.2. Clinical and Neuropsychological Measures

At admission, demographic and clinical data were collected from all the patients (experimental and control groups). Duration of coma was calculated according to [26,27] (readers should note that an update was published after the beginning of this trial, and they should now refer to Giacino et al. [28]). Before the administration of the rehabilitation program (T0), we also collected scores at the clinical and behavioral scales: *Glasgow Outcome Scale* (GOS), *Glasgow Outcome Scale Expanded* (GOS-E), and *Levels of Cognitive Functioning Assessment Scale* (LCF). Among the motor-functional measures, we recorded the *Disability Rating Scale* (DRS) and the *Functional Independence Measure* (FIM) scores. We also acquired the Intelligence Quotients (IQs), by administering the *Wechsler Intelligence Scale for Children-III* (WISC-III) or the *Wechsler Adult Intelligence Scale-Revised* (WAIS-R), according to the patients’ age. The full assessment was repeated at the end of the rehabilitation program. See Supplementary Table S1 for a brief description of each scale, and for the related bibliography.

### 2.3. Psychometric Evaluation (Primary Measures)

Each patient received initial psychometric evaluation before entering the rehabilitation program (T0). After four weeks (T1), the evaluation was repeated, enabling the assessment of possible changes in the psychometric levels. At both time points, the patients were administered the *Rey-Osterrieth complex figure test* (REY), the test for *immediate and delayed memory of a list of words* of the Italian “Batteria di Valutazione Neuropsicologica” (BVN) battery (BVNLi and BVNLd, respectively), the test for *immediate and delayed recall in prose memory* of the Italian BVN battery (BVNPi and BVNPd), the test for *immediate and delayed recall in positional memory supra-span* (SUPRASPANi and SUPRASPANd), and the Italian *TEMA test for the learning of couples of related and unrelated words* (TEMA) (see Supplementary Table S2 for a brief description of each tool, and for the related bibliography).

Physio-kinesis/occupational evaluations were performed at the end of each treatment session by therapists, who reported on a standard form the number of repetitions each patient needed to learn a given procedure (see Supplementary Material S3).

### 2.4. Theoretical Framework for the Pediatric Adaptation of Rehabilitation Programs

IM-FTP aims to be a pediatric homologous of memory-focused interventions for adults. Mere adaptation of the difficulty of the proposed exercises was needed. However, this was insufficient to fully take into account the difference between the two populations. We specifically worked on the delivery modality (requests and procedures), by acting on three elements:

(1) The developmental needs of the child (need to play, need of novelty, acceptance of the challenge and of the failure/mistake, need to understand their own difficulty while overcoming frustration and avoiding giving up).
(2) The different cognitive functioning compared to the adult. The child approaches tasks in ipo-strategic and impulsive manner, due to the immaturity of the executive functions. Also, they can only deploy reduced wealth of experience and limited procedure automation.
(3) The immature self-motivation and self-determination, which negatively impact on the compliance to the rehabilitation work.

The metacognition content was delivered through play. Cartoons/vignettes, role playing and familiar videogames were incorporated in the delivery.

### 2.5. Rehabilitation Program

For both experimental and control groups, the rehabilitation program consisted of an intensive treatment lasting 4 weeks, and including 3 daily interventions each working day (5 working days per week, for a total of 20 days of treatment and 60 sessions). The 3 daily sessions were organized as follows: 1 physio-kinesis and/or occupational therapy, 1 speech therapy, and 1 neuro-psychology treatment. Each session lasted 45 min.

The rehabilitation assignments were initially selected from sets of pre-defined exercises, after considering the age of each patient and the extent of their motor and/or cognitive damage. The selection was adapted throughout the treatment, on a session-by-session basis.

#### 2.5.1. Intensive Memory-Focused Training Program (IM-FTP)

Patients in the experimental group entered the IM-FTP. During each session, the patient was engaged in a one-to-one interaction with the therapist. Overall, the treatment targeted the following specific memory functions: verbal and visuo-spatial short-term memory, verbal and visuo-spatial long-term memory, working memory and procedural memory. During the speech and neuro-psychology treatments, the therapist used both paper-based worksheets and dedicated software. Each session required completion of pre-determined exercises and the difficulty level was calibrated on a trial-by-trial basis, to work in close proximity to the patient’s personal limit. During the physio-kinesis/occupational therapy the staff administered exercises both requiring and not requiring equipment, as well as floor routines. Equipment mainly consisted of clothing, small cubes, hoops, pillows, personal toothbrush and a coffee-pot. The treatment protocol was organized and administered as follows.

*Physio-kinesis/occupational therapy*. The session consisted of targeted stimulation of the procedural memory for 45 min. This was initially done by organizing the subsequent postural steps for reaching a standing position: the patient entered the procedure by sitting on a rug, and he/she was guided to stabilization in an upright posture. Then the therapist proposed a guided step-by-step procedure for getting undressed. Patients were challenged to construct a tower or pyramid of blocks, followed by ergotherapy (therapy using tool manipulation and procedures directed to a goal/final result), motor sequences involving the upper limbs, and exercises based on manipulation of interlocking and snapping objects, and assembling of jigsaws. Patients were also challenged in the lower limb motor skills by following a trail composed of various obstacles and surfaces. The session ended with the execution of the procedure for preparing coffee in a coffee-pot and for brushing teeth. To accommodate the patients’ different grades of impairment and time needs, the therapists were left free to select a subset (at least 3) out of the 7 standardized procedural exercises at any session. Single patients’ trajectories of performance during the 20 sessions are reported in Supplementary Material S3.

*Neuro-psychology treatment*. During the neuro-psychology treatment, patients were individually paired with a therapist for the whole duration of their treatment. The rehabilitation protocol included two levels of interventions: memory function-specific training and memory meta-cognitive support to treatment. Memory function-specific training targets the various aspects of visuo-spatial memory (short, long term and working memory) by using specific software in their native language and pen and pencil exercises. The use of meta-cognition as a support tool to treatment was designed to increase a patient’s self-reflection, awareness, control of their cognitive function through understanding of how different components of memory work, and compliance to treatment. This is achieved through the deployment and practice of strategies to increase control of impulsivity, self-monitoring ability, and the coding and recalling abilities, and by constant feedback from the therapist, in accordance to clinical neuropsychology best practice. Each session was subdivided into 3 blocks, lasting 15 min each:

(1) Stimulation of the short-term visuo-spatial memory. We trained the patient to work around their specific deficit and discover strategies for memorizing through visualization, acting on the patient’s short-term memory through empowerment. We introduced the concept of an “internal camera” for memorizing selected images and situations [29], and we personalized the training through a selection of activities to be continuously adapted to the patient’s need. Then patients solved logical matrices and visual memory exercises taken from the Test of Visual Perception Skills v.3 (TVPS-3) protocol [30], and exercises of object permanence.
(2) Stimulation of the long-term visuo-spatial memory. In this block the therapist administered exercises of visual memory with interference, and training exercises from the TVPS-3 protocol. Then, exercises were carried out, divided in sections, to train the recall of image series, recall of object position inside a scenery, and learning of simple visuo-spatial sequences [29]. Each section administered exercises grouped according to type and graded according to increasing difficulty. Then the therapist carried out exercises for the memorization of 3 objects and their respective spatial location; the task was followed by the patient’s involvement in a distracting skill, and the subsequent request to recall the positions of the objects and to find them inside the room. Lastly, patients were administered exercises of supra-span visual memory.
(3) Stimulation of the visual working-memory (visuo-spatial notepad). In this block the staff administered n-back exercises, Sudoku, solo card games (e.g., Spider and similar), updating exercises and exercises from Marzocchi et al. [31], which includes different visual and audio activities, aiming at the development and empowerment of working-memory.

*Speech treatment*. During the speech treatment patients practiced short-term, long-term and verbal working memory with specific exercises. With the therapist’s help, they also reflected on the way their cognitive processes work and on how these could be addressed. The session was subdivided into 3 blocks, lasting 15 min each:

(1) Stimulation of the short-term verbal memory. Repetition exercises (continuous reiteration of a given span of numbers or words) were initially administered, based on Rudland et al. [29]. Then the therapist administered memory timing exercises [32,33]. Patients were requested to remember numbers and words for short time (e.g., store and recall, by typing a phone number or writing a car number plate).
(2) Stimulation of the long-term verbal memory. Patients were asked to recall information, also through the method in Powell, Gollin et al. [32,33], lists of words, newspaper articles, names and numbers. Then, exercises of text recall with and without interference were delivered, tasks for the learning and retention of words lists, tasks for learning and retention of couples of linked and non-linked words, and exercises for face-name association.
(3) Stimulation of the verbal working-memory. Therapists administered logical grids, the Paced Auditory Serial Addition Task and the Children’s Paced Auditory Serial Addition Task [34,35], exercises on acronyms, and tasks for the memorization of the first, second or third element contained in a sentence. Additionally patients took on tasks of the generation of sentences for learning couples of words.

#### 2.5.2. Standard Training Program

Patients in the control group took part in a standard rehabilitation program: they attended physio-kinesis, neuropsychological and speech sessions daily, with the same schedule, overall duration and intensity of the experimental group. Each patient was paired with a therapist, who proposed pen-and-pencil exercises, computer games and role-playing, analogously to what was administered to the experimental group during IM-FTP. Nevertheless, in this program, the requests were designed to stimulate different cognitive domains (motor, attention and executive functions), simultaneously or sequentially, without specific focus on memory. Different professionals (one physiotherapist, one neuropsychologist and one speech therapist) delivered each type of treatment. Therapists were the same in both arms of the study. Clinical measures and psychometric evaluations were performed by different assessors from the therapists involved in treatment. The difference between IM-FTP and standard programs is in the *focused* vs. *unfocused* nature of the treatment. This fact provides rationale for the comparison between the experimental and control groups after treatment. Participants in the healthy group received no treatment.

### 2.6. Statistical Analysis

Continuous data is presented as means, standard deviations (SDs) and percentages; discrete data is presented as medians and modes. Between-group variances are compared through Levene’s test. Binary and ordinal data are compared through χ^2^ test. *t*-test for differences in means is applied to normally distributed within-group dependent samples; Wilcoxon Signed-Rank test for difference in medians is performed in order to compare within-group dependent samples non-parametrically, before vs. after the rehabilitation treatment (i.e., at time-point T0 vs. T1). Analysis of covariance (ANCOVA) is applied to test group effects between the experimental and control groups during rehabilitation. As Levene’s test has ruled out heterogeneity of variances for all the randomization variables, we only need to correct for the (unbalanced) psychometric tests at T0. For this reason, one ANCOVA test has been applied to each psychometric evaluation at T1, with the unique covariate being the corresponding psychometric score before the initiation of the rehabilitation treatment (T0). As this is a feasibility trial, correction for multiple comparisons was not applied. Rather, sample size estimation was performed to plan a powered randomized clinical trial (alpha = 0.05; power = 0.9).

### 2.7. Magnetic Resonance Imaging (MRI) Acquisition and Functional MRI Analysis

Patients in the experimental group underwent brain MRI before and after treatment. Participants in the healthy group underwent one MRI session only. MRI acquisition was performed using a 3.0 T imaging scanner (Philips Medical Systems, Best, the Netherlands). Resting state functional MRI (rsMRI) scans were acquired using a T2 *-weighted single-shot echo-planar imaging sequence during rest. In addition, we acquired a three-dimensional (3D) T1-weighted Turbo-Field-Echo sequence and a T2-weighted Turbo-Spin-Echo sequence. Details of MRI findings, MRI sequences and the processing pipeline applied to the rsMRI scans are described in Supplementary Material S4.

## 3. Results

### 3.1. Study Sample

Twenty-one patients were enrolled in the study. Eleven (5 males) were assigned to the experimental group; 10 (6 males) entered the control group. In both groups, traumatic brain injury was the prevalent etiology. Mean age at injury was 12.2 years in the experimental group and 13.8 years in controls. Average days of coma were 16.9 in the experimental and 28.6 in the control groups. In both groups, patients were hemiparetic for the most part. Diffuse axonal injury and multifocal brain damage were found in most of patients’ structural MRI scans. Demographic, clinical characteristics and Intelligence Quotient at admission are reported in Table 1. Seven children (4 males; mean age = 13.9) entered the healthy group and received one neuroimaging session (T0 only). As the study targeted in-patients and no adverse events were observed, we registered no drop-out. Three sessions had to be rescheduled to a time of the day different than the one initially planned, or to another day of the same week. One patient suffered cephalgia during the treatment period, with the same frequency as before enrollment, and could however attend all the sessions.

### 3.2. Clinical and Behavioral Scores before and after Rehabilitation

At presentation in the acute hospital, GCS score was 5 for the experimental group and 6, on average, for the control group. Clinical and behavioral scores at admission to the rehabilitation center, and after one-month treatment with IM-FTP are summarized in Table 2. Group results and the statistical analyses of the within-group comparison before and after treatment (T0 vs. T1) show that both groups improved in all the clinical scores during the one-month period of study. Between-group comparisons provided no statistical significance at T0, as well as at T1, thus indicating that the experimental and control groups were initially balanced for clinical severity, and remained below statistical significance in average clinical scores after treatment (*p* > 0.05 for all tests). Overall, the clinical improvement of the two groups was comparable over the one-month period.

### 3.3. Neuropsychological Scores before and after Rehabilitation

Table 3 shows the scores of the neuropsychological tests before and after treatment, for the experimental and control groups. After 4 weeks of intensive IM-FTP, the experimental group showed significant increase in performance at the BVNPi (*p* = 0.043), and BVNPd increased up to the normative levels (0.029). This represents clear improvement in both immediate and delayed recall prose memory. Significant increase in BVNLi scores (*p* = 0.045), but not in BVNLd (*p* > 0.05), indicates improvement in the immediate, but not delayed, learning of lists of words. This improvement in immediate verbal learning was also supported by the significant increase of TEMA z-scores (*p* = 0.002), which indicates successful learning of semantically related and unrelated words. On the other hand, increases in SUPRASPANi and SUPRASPANd z-scores could not be confirmed (*p* > 0.05), despite an overall trend of improvement being observed. Additionally, REY z-scores increased (*p* = 0.016), despite remaining clearly below the normative, meaning partially improved visual recall. Overall, these results indicate that visuo-spatial memory was the domain least responsive to treatment.

After 4 weeks of intensive standard rehabilitation, controls showed increased scores at BVNLi (*p* = 0.001) and BVNLd (*p* = 0.003), indicating improvement in the immediate and delayed learning of lists of words. The group also had improvement at TEMA z-scores (*p* = 0.021), indicating better learning of semantically related and unrelated words. Increases in BVNPd and REY could not be confirmed (*p* > 0.05), despite a trend of improvement being observed. The remaining tests did not show any improvement.

Between-group comparisons of means and medians before treatment showed that the experimental and control groups were initially comparable at all neuropsychological scores but TEMA, with higher initial TEMA median score for the experimental group (*p* = 0.030). ANCOVA after treatment showed differences at TEMA (F = 21.30 and *p* = 0.001 for the group effect) and at BVNPi (F = 8.89 and *p* = 0.015 for the group effect), with higher estimated marginal means of improvement for the experimental group, indicating better learning of semantically related and unrelated words and larger improvement in immediate recall in prose memory.

Through the physio-kinesis and occupational therapy sessions, one patient fully recovered the ability to stand up from the floor, from bottom score, in 6 sessions. Ergotherapy and a trail path were administered to all patients in at least one session. Preparing a coffee pot and brushing the teeth also showed fast improvements overall. Undressing and the pyramid showed varying recovery times (Supplementary Material S3).

To plan a full randomized trial, calculation of the sample size for each neuropsychological tool is provided in Table 4. Sample size equal to 31 is needed in order to obtain a powered trial for all the measures. Computation of the effect size and observed power from the available sample size is reported in the Supplementary Table S5.

### 3.4. Resting State Functional MRI (rsMRI)

**Figure 1:**
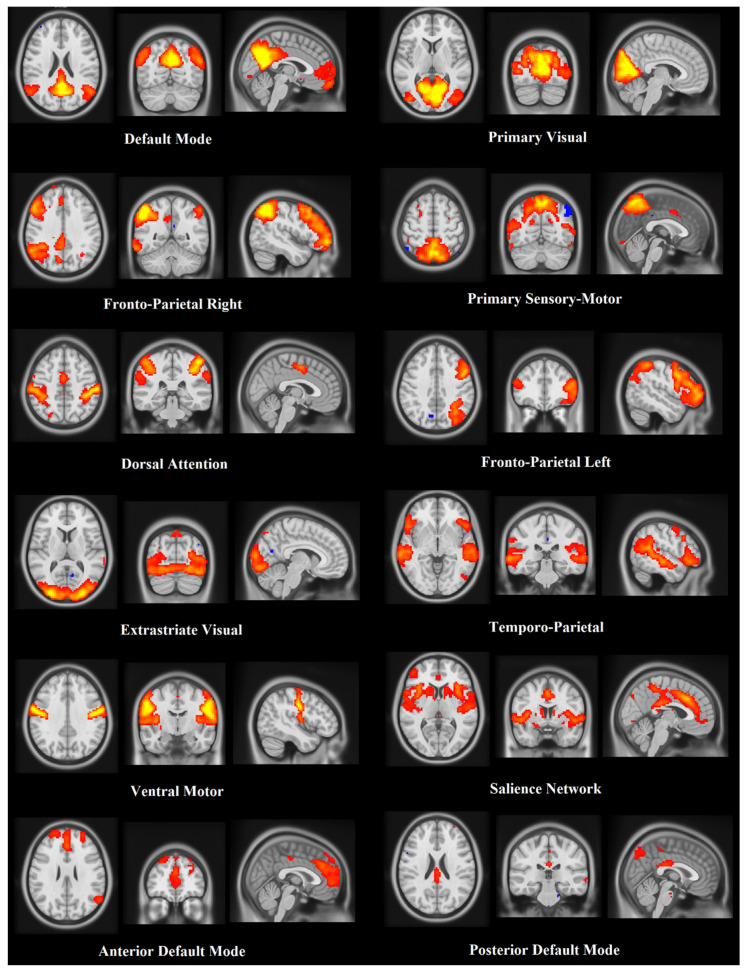
ICA components resulting from rsMRI processing through MELODIC. Results refer to patients in the experimental group. The analysis isolated three components of the default mode network: an overall default mode, one mainly anterior and one mainly posterior component (bottom of figure). The primary visual and an extrastriate visual components were also isolated, as well as the dorsal attention and salience networks. One component captured a well-defined motor network. The fronto-parietal network was split into one right, one left and one temporal component.

**Figure 2:**
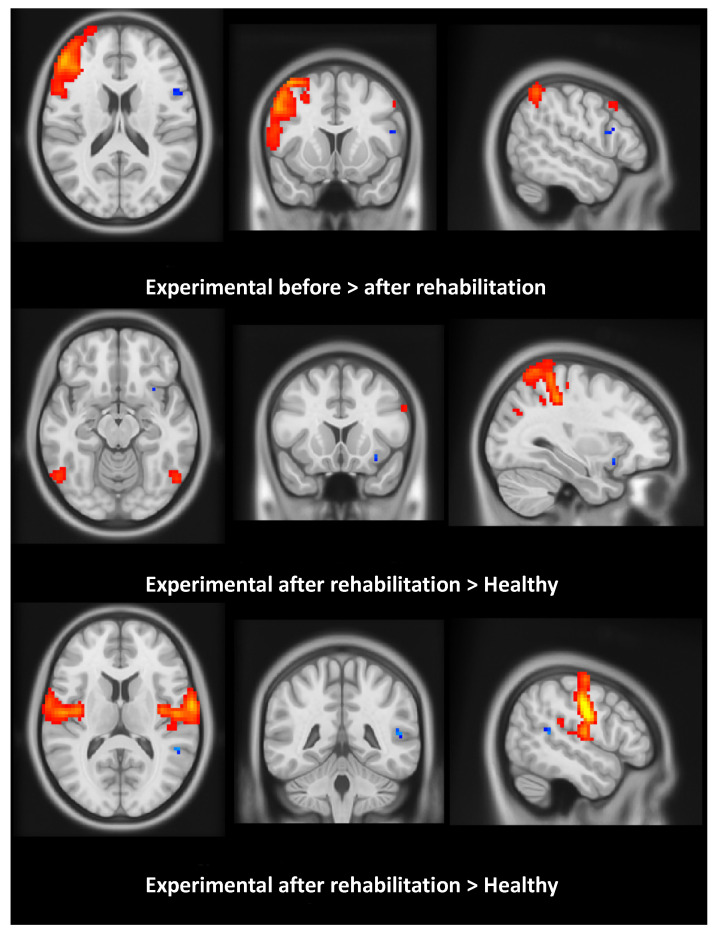
rsMRI processing. Results refer to patients in the experimental group, and to the condition “before” vs. “after” rehabilitation. Red areas correspond to the selected network activation, while blue voxels represent significantly different clusters of activity. In the sensory-motor network, involvement of the left insula and left superior temporal cortex was evident when comparing the experimental after rehabilitation versus the healthy group. In the right fronto-parietal network, we found involvement of the left frontal inferior cortex when comparing the experimental group before and after rehabilitation.

To test for any effect of IM-FTP on brain plasticity, rsMRI data were acquired from the experimental group before and after treatment, and from healthy group for reference. An Independent Component Analysis (ICA) was performed through Multivariate Exploratory Linear Optimized Decomposition into Independent Components (MELODIC) in order to extract significant brain networks and test differences in functional connectivity related to the rehabilitative intervention (see Supplementary Material S4). Twelve ICA components/networks were selected and further analyzed to extract networks (Figure 1). For both intra-network and inter-network functional connectivity analysis, we compared: (1) healthy versus experimental groups before rehabilitation (BR); (2) healthy versus experimental after rehabilitation (AR); and (3) experimental BR versus experimental AR. Considering the sensory-motor network, a major involvement of the left insula and left superior temporal cortex was evident when comparing experimental AR versus healthy (experimental AR > healthy). In the right fronto-parietal network, a major involvement of the left frontal inferior cortex was evident when comparing experimental BR to experimental AR (experimental BR > experimental AR). Significant difference clusters are shown in Figure 2, superimposed on the corresponding group-average networks. For the inter-network functional connectivity, we found a significant (*p* = 0.036 corrected for multiple network comparison) difference between the primary visual network and the temporo-parietal network, when we compared experimental BR and healthy (experimental BR > healthy). No significant differences were found for all the other comparisons.

## 4. Discussion

This paper describes the design and implementation of an intensive rehabilitation intervention focused on the recovery of memory after severe ABI in the school-age. We report results about the administration of this intensive program, called IM-FTP, to eleven pediatric patients with ABI. We compare results with those obtained from a control group of ten patients receiving a standard non-focused intervention of equal intensity for the recovery of higher cognitive functions. Both groups improved their clinical and behavioral scores throughout the rehabilitation path, thus confirming the efficacy of the two interventions.

The group receiving IM-FTP showed significant improvement in immediate and delayed recall of a short story. This was not observed in the group receiving the standard treatment. Also, IM-FTP significantly improved performance at the Rey-Osterrieth complex figure test, which showed non-significant trend of improvement after the standard treatment. Both interventions, IM-FTP and standard, enabled significant improvement in the recall of couple of words (TEMA) and immediate recall of list of words, but only the standard program demonstrated significant recall of list of words delayed in time. Overall, this hints at the improved ability of IM-FTP to successfully address and train recall abilities when challenged in a semantic context, either verbal or visuo-spatial. Previous work involving adults with mild TBI showed that, in contrast with the recognition processes involved in visual memory, recall processes seem to be more vulnerable to damage [36]. Consequently, IM-FTP could add specificity, and thus precision, when targeting the recall abilities.

Neuroimaging showed abnormalities in the primary visual and in the temporo-parietal networks of patients entering IM-FTP. This was expected, due to the memory impairment [37]. After IM-FTP, functional modification in the left frontal inferior cortex was observed, which we interpreted as both plastic adjustment and compensatory effect [38]. The left inferior frontal gyrus, in close relationship with the left posterior middle temporal gyrus, plays an essential role in syntactic analysis [39] and lexical selection [40], and is one of the areas stimulated during training with IM-FTP.

Previous literature addressing memory rehabilitation in children offers few pilot studies [8,10,41,42,43], all conducted at least one year after injury, longer in duration but less intense than IM-FTP. To our knowledge, this is the first study focusing on the sub-acute phase of disease; patients received one cognitive evaluation and started treatment three months after the acute event, on average. Our study is also the largest to date, despite the group sizes being still small for robust statistical analysis, and the first to introduce neuroimaging for the assessment of memory treatment efficacy in pediatric ABI.

In our study we aimed to design and deliver a rehabilitation intervention with specificity to one cognitive function. Based on a hierarchical cognitive model, rehabilitation should initially focus on the basic and high-level components of attention [44], which are the underlying foundation of all the other cognitive skills [45,46]. Following the stabilization of attention, specific memory functions have to be selectively stimulated, according to a common protocol, but with specificity to each rehabilitation setting and through holistic approach to the patient [47,48,49,50,51,52]. Particularly, if proved with longer follow-ups, the “strategy” hypothesis could imply that, in the early sub-acute phase, in which a status of functional dis-organization due to executive deficits persists, rehabilitation can focus on the teaching of effective learning strategies that are inexpensive to implement. This might be an effective preliminary step to a subsequent stage of recovery, characterized by higher functional selectivity. The intervention proposed here is designed to present increasing levels of difficulty, which allow adaptation of the proposed activities to single patient’s capabilities, and it is not age-specific. This results in a highly structured and flexible rehabilitation program: the structure enables the comparison of patients’ performance, while the flexibility allows adaptation to the patients’ specificity, age and recovery trajectory.

### Limitations

This feasibility study should be interpreted while considering a number of limitations. Firstly, the small sample size and the heterogeneity of etiologies might have jeopardized the statistical significance of some comparisons. For example, despite that the days of coma were not significantly different between groups, means show that the control group had longer duration than the experimental overall. These group differences, although not significant, may account for some of the differences between groups on memory testing. Analogously, baseline psychometric test scores for the control group had slightly lower z scores than the experimental on 5 of the 7 measures; this is a potential source of bias. Secondly, spontaneous recovery, still occurring in the post-acute phase, certainly played a role, and an equal effect for the two groups (experimental and control) was assumed—but not proved—in this study. Unfortunately, we could not set up an untreated arm, due to ethical implications. However, as already observed for AMAT-c, the fact that patients improved despite the heterogeneity of diagnosis could be supportive of program external validity [7]. Further testing of this training program in larger groups, as well as assessment of the transfer of improvement to the patient’s everyday lives in school and at home, are needed, however. Thirdly, between-group comparisons after rehabilitation suggest that both IM-FTP and standard interventions were effective, in line with the holistic rehabilitation standards offered by our center; however, the partial recovery/improvement of specific mnemonic functions after both interventions might advocate for longer treatment periods. Fourthly, the assessment of procedural memory performance during physio-kinesis therapy was quantitative but was performed through an unpublished and non-validated checklist. This point will be amended before progressing to the powered trial. Penultimately, this feasibility study did not include specific measures of satisfaction and acceptability of the treatment, which will be introduced in the powered trial in the form of a questionnaire.

Lastly, this work raises a few practical considerations. Therapists provided daily feedback to parents, and remained unchanged throughout the program. This may have helped children in building their self-confidence with the tasks proposed during the rehabilitation intervention. On an economical basis, the intervention is intensive but short (1 month) and it does not make use of expensive equipment. Thus it is compatible with the resources of most national healthcare systems.

## 5. Conclusions

We aim to provide a detailed description of a multidisciplinary memory-focused rehabilitation protocol, IM-FTP, based on holistic multidisciplinary approach and continuous therapeutic support to the individual in school-age. Considering current literature [43], we designed a structured yet flexible intervention, which can be adapted to single patients by preserving comparability between them, and which can be proposed up to few months after injury. We defined the timing, activities and exercises to be proposed in the different contexts of intervention (neuropsychological, speech and physio-kinesis therapies). We described the implementation details in this article to maximize reproducibility by other centers, and we tested it on larger groups then those reported in previous comparable studies. The description of the rehabilitation activities, procedures, materials and tools fills a gap in the present scientific literature. IM-FTP now needs to be tested in a powered clinical trial, before being used as a model of intervention by staff who deal with patients with ABI in the school age.

## Figures and Tables

**Table 1:** Clinical and demographic characteristics and intelligence quotient of the sample at admission.

Total Sample (n = 21)	Experimental Group (n = 11)		Control Group (n = 10)		Test
	N	%	N	%	*χ*^2^ (*p*-Value)
Gender					
Male	5	45.4	6	60.0	0.44 (0.505)
Female	6	54.5	4	40.0	
Etiology					
Traumatic Brain Injury (TBI)	7	63.7	7	70.0	0.10 (0.757)
Non Traumatic Brain Injury (NTBI)	4	36.3	3	30.0	
of Which:					
Ictal Stroke	2			1	
Hemorrhagic Stroke	1			1	
Immune Encephalitis	1			1	
	**Mean**	**SD**	**Mean**	**SD**	***Wilcoxon*** **(*p*-value)**
Age at Injury [months]	146.4	41.4	165.9	27.6	1.41 (0.159)
Age at Admission [months]	148.6	42.8	169.4	29.3	1.33 (0.184)
Days of Coma	16.9	14.2	28.6	24.4	1.17 (0.244)
Days from Injury/Illness to Treatment	30.1	14.6	35.1	18.9	0.44 (0.503)
	**N**	**%**	**N**	**%**	***χ*^2^** **(*p*-value)**
Need of Neurosurgery	5	45.5	3	30.0	0.53 (0.466)
Epileptic Seizures	1	9.1	0	0.0	0.96 (0.329)
Motor Impairment					
Quadriparesis	0	0.0	2	20.0	3.96 (0.266)
Hemiparesis	7	63.6	7	70.0	
Ataxia	1	9.1	0	0.0	
None	3	27.3	1	10.0	
Brain Lesions at Magnetic Resonance Imaging					
Diffuse Axonal Injury	4	36.3	5	50.0	2.07 (0.356)
Multifocal Damage	5	45.5	5	50.0	
Damage Due to Extradural Hematoma	2	18.2	0	0.0	
	**Mean**	**SD**	**Mean**	**SD**	***Wilcoxon*** **(*p*-value)**
Wechsler Intelligence Scale for Children or Wechsler Adult Intelligence Scale					
Total Intelligence Quotient	67.8	15.4	60.5	13.7	1.09 (0.275)
Verbal Intelligence Quotient	79.7	16.8	79.9	16.1	0.01 (0.998)
Performance Intelligence Quotient	66.0	17.8	66.5	20.2	0.08 (0.940)

Experimental and Control groups are reported in separate columns. The two groups were balanced for gender, and the main etiology was TBI. Days from illness to treatment show that children were in the post-acute phase of the disease at enrollment. In both groups few patients had needed neurosurgery, and in the experimental group one patient had developed pharmacologically controlled epileptic seizures. The most common motor impairment was hemiparesis in both groups; however, the experimental group had 3 patients with no motor impairment, while the control group had only one, and two quadriparetic children. Brain lesions at MRI were comparable in the two groups. WISC-III and WAIS-R were comparable in the two groups, although below the scores in the healthy age-matched population.

**Table 2:** Scores at the clinical and behavioral scales, before (T0) and after (T1) rehabilitation. Experimental and Control groups are reported in separate columns.

	Experimental Group (n = 11)					Control Group (n = 10)				
	T0		T1		Statistics	T0		T1		Statistics
	Median	Range	Median	Range	*Wilcoxon* (*p*-Value)	Median	Range	Median	Range	*Wilcoxon* (*p*-Value)
**GCS Score**	5	3–8	-	-		6	3–8	-	-	
**GOS Score**	3	2–4	4	3–5	2.4 (0.015) *	3	2–3	4	3–5	2.6 (0.010) *
**GOS-E Score**	3	2–5	6	4–7	2.8 (0.004) *	3	2–4	6	4–7	2.8 (0.005) *
**LCF Score**	6	2–8	8	7–8	2.7 (0.007) *	5	2–7	8	8	2.8 (0.005) *
	**Mean**	**SD**	**Mean**	**SD**	**t (*p*-value)**	**Mean**	**SD**	**Mean**	**SD**	**t (*p*-value)**
**DRS**	13.2	5.2	4.6	1.8	5.8 (<0.001) *	15.8	6.6	4.2	1.8	5.3 (0.001) *
**FIM**	35.7	24.4	78.0	30.9	4.6 (0.001) *	36.3	28.6	97.5	22.5	6.8 (<0.001) *

* significant at *p*-value < 0.05. GCS: Glasgow Coma Score; GOS: Glasgow Outcome Scale; GOS-E: Glasgow Outcome Scale Extended; LCF: Levels of Cognitive Functioning Assessment Scale; DRS: Disability Rating Scale; FIM: Functional Independence Measure.

**Table 3:** Scores at the neuropsychological tests, before (T0) and after (T1) rehabilitation. Experimental and Control groups are reported in separate columns. Abbreviations indicate the Rey-Osterrieth complex figure test (REY), the test for immediate and delayed memory of a list of words of the Italian BVN battery (BVNLi and BVNLd, respectively), the test for immediate and delayed recall in prose memory of the Italian BVN battery (BVNPi and BVNPd), the test for immediate and delayed recall in positional memory supra-span (SUPRASPANi and SUPRASPANd), and the Italian TEMA test for the learning of couples of related and unrelated words (TEMA).

	Experimental Group (n = 11)					Control Group (n = 10)				
	T0		T1		Statistics	T0		T1		Statistics
	Mean z-Score	SD	Mean z-Score	SD	t (*p*-Value)	Mean z-Score	SD	Mean z-Score	SD	t (*p*-Value)
**REY**	−2.8	1.3	−2.4	1.9	2.8 (0.016) *	−3.0	1.3	−2.5	2.2	1.3 (0.119)
**BVNLi**	−2.5	2.1	−1.9	2.2	1.9 (0.045) *	−3.3	1.6	−1.9	2.4	5.3 (0.001) *
**BVNLd**	−3.0	2.2	−2.3	2.7	1.4 (0.103)	−4.1	3.0	−2.9	3.7	4.2 (0.003) *
**BVNPi**	−1.8	2.5	0.5	0.5	2.3 (0.043) *	−2.0	2.6	−2.2	2.0	0.2 (0.431)
**BVNPd**	−1.6	2.8	0.0	1.3	2.6 (0.029) *	−1.2	2.4	−0.7	1.3	1.4 (0.108)
**SUPRA SPANi**	−2.9	2.8	−2.3	3.4	1.4 (0.105)	−3.3	2.0	−3.7	1.9	0.4 (0.341)
**SUPRA SPANd**	v2.4	2.2	−1.9	2.3	1.2 (0.137)	−2.0	2.4	−2.9	1.6	0.2 (0.436)
	**Median**	**Range**	**Median**	**Range**	***Wilcoxon*** **(*p*-value)**	**Median**	**Range**	**Median**	**Range**	***Wilcoxon*** **(*p*-value)**
**TEMA**	12	9–29	21	14–30	2.8 (0.002) *	7	1–13	12	5–16	2.0 (0.021) *

* difference was significant at *p*-value < 0.05; BVN: Batteria di Neurovalutazione Psicologica; TEMA: Test of memory and Learning.

**Table 4:** Calculation of the sample size for the neuropsychological tools. Alpha is set to 0.05 and power at 0.9.

	Sample Size
REY	6
BVNLi	31
BVNLd	14
BVNPi	7
BVNPd	11
SUPRASPANi	26
SUPRASPANd	27
TEMA	3

## Supplementary Materials

The following are available online at https://www.mdpi.com/2076-3425/10/7/430/s1, Document: Supplementary material. Supplementary Table S1: Clinical scales, Supplementary Table S2: Psychometric tests, Supplementary Material S3: Physiotherapy procedures and single patient results; Supplementary Material S4: Radiological methods and findings; Supplementary Table S5: Power analysis for the clinical trial.
