# Supplementary material for: Feasibility Randomized Trial for an Intensive Memory-Focused Training Program for School-Aged Children with Acquired Brain Injury
Source: Brain Sci. 2020 Jul 7;10(7):430. doi: 10.3390/brainsci10070430 (PMC7407971; doi:10.3390/brainsci10070430)
Supplement: Supplementary file 1 [file brainsci-10-00430-s001.pdf]

Supplementary Table 1

Clinical scales applied in the present study. From left to right: name and reference to the main bibliography, abbreviation, brief description of the scale.

NAME and REFERENCE	ABBREVIATION	DESCRIPTION
GLASGOW COMA SCALE[1]	GCS	The *Glasgow Coma Scale* is the most common scoring system used to describe the level of consciousness in a person following a traumatic brain injury. The GCS is scored between 3 and 15, 3 being the worst, and 15 the best. It is composed of three parameters: Best Eye Response, Best Verbal Response, and Best Motor Response.
GLASGOW OUTCOME SCALE[2]	GOS	The *Glasgow Outcome Score* applies to patients with brain damage allowing the objective assessment of their recovery in five categories: death, persistent vegetative state, severe disability, moderate disability and low disability. This allows a prediction of the long-term course of rehabilitation to return to everyday life.
GLASGOW OUTCOME SCALE – EXTENDED[3]	GOS-E	The *Extended GOS* provides more detailed categorization, with respect to GOS, into eight categories by subdividing the categories of severe disability, moderate disability and low disability (i.e. good recovery) into a lower and upper category each.
LEVELS OF COGNITIVE FUNCTIONING ASSESSMENT SCALE[4]	LCF<br>(or LCFS or LOCFAS)	The *Rancho Level of Cognitive Functioning Scale* is used to assess cognitive functioning in post-coma patients. It was developed for use in the planning of treatment, tracking of recovery, and classifying of outcome levels. Use of the scale generates a classification of the patient in one of eight levels: No response; Generalized; Localized; Confused-agitated; Confused, inappropriate, non-agitated; Confused-appropriate; Automatic-appropriate; Purposeful-appropriate.
DISABILITY RATING SCALE[5]	DRS	The *Disability Rating Scale* evaluates juvenile and adult individuals with moderate and severe traumatic brain injury in inpatient rehabilitation settings. DRS can track an individual from coma to community. DRS measures across a wide span of recovery items: eye opening, communication ability, motor response, cognitive ability for "Feeding," "Toileting" and "Grooming". The maximum score a patient can obtain on the DRS is 29 (extreme vegetative state). A person without disability would score zero.
FUNCTIONAL INDEPENDENCE MEASURE[6]	FIM	The *Functional Independence Measure* evaluates performance to standard criteria of self-care, sphincter control, transfers, locomotion, communication, and social cognitive tasks in individuals; it allows thorough evaluation of functional independence by concurrently comprising the motor, cognitive and social domains. Scores adaptation, with respect to normative data, is applied for young age children (Wee-FIM).
WECHSLER INTELLIGENCE SCALE FOR CHILDREN[7]	WISC	The *Wechsler Intelligence Scale for Children* is a standardized measure of intelligence quotient. This instrument provides a Full Scale Intelligence Quotient (FSIQ) and four Composite or Index Scores:<br>1. The Verbal Comprehension Index (VCI), tapping into verbal memory and reasoning skills;

		2. The Perceptual Reasoning Index (PRI), measuring visual information processing and non-verbal reasoning;<br>3. The Working Memory Index (WMI), having attention and concentration components;<br>4. The Processing Speed Index (PSI), derived from the scores of two subtests that require quick mental processing.<br>Scores are provided as standard scores with a mean of 100 and a standard deviation of 15. The scale is applied up to 16 years of age.
WECHSLER ADULT INTELLIGENCE SCALE[8,9]		The *Wechsler Adult Intelligence Scale* is a standardized measure of intelligence quotient for people older than 16 years.<br>Analogously to the WISC, this instrument provides a Full Scale Intelligence Quotient (FSIQ) and four Composite or Index Scores:<br>1. The Verbal Comprehension Index (VCI), tapping into verbal memory and reasoning skills;<br>2. The Perceptual Reasoning Index (PRI), measuring visual information processing and non-verbal reasoning;<br>3. The Working Memory Index (WMI), having attention and concentration components;<br>4. The Processing Speed Index (PSI), derived from the scores of two subtests that require quick mental processing.

Bibliography

Formatted: French (France)

1. Teasdale, G., & Jennett, B. (1974). Assessment of coma and impaired consciousness. A practical scale. *Lancet*, 2(7872), 81-4.

2. Jennett B, Bond M. (Mar 1, 1975). "Assessment of outcome after severe brain damage.". Lancet **1** (7905): 480–484.

3. Wilson JTL, Pettigrew LEL, Teasdale GM. Structured in terviews for the Glasgow Outcome Scale and the Extended Glasgow Outcome Scale: Guide lines for Their Use. J Neurotrauma 15(8): 573-85. 1997.

4. Flannery J, Korcheck S. Use of the levels of cognitive functioning assessment scale (LOCFAS) by acute care nurses. *ApplNurs Res* 1993;6:167-9.

5. Hall K, Cope DN, Rappaport M. Glasgow Outcome Scale and Disability Rating Scale: comparative usefulness in following recovery in traumatic head injury. *Arch Phys Med Rehabil* 1985;66:35-7

6. Wright J. The FIM (TM). The Center for Outcome Measurement in Brain Injury. 2000.

7. Wechsler D. (1949). *Wechsler intelligence scale for children*. New York: The Psychological Corporation.

8. Wechsler, David (1939). The Measurement of Adult Intelligence. Baltimore (MD): Williams & Witkins.

9. Wechsler, D. (1958). The Measurement and Appraisal of Adult Intelligence (4th ed.). Baltimore (MD): Williams & Witkins.

Supplementary Table 2

Psychometric tests applied in the present study. From left to right: name and reference to the main bibliography, abbreviation, brief description of the test.

NAME and REFERENCE	ABBREVIATION	DESCRIPTION of the tested SKILLS
REY-OSTERRIETH COMPLEX FIGURE TEST (REY MEMORY TEST)[1]	REY	Ability to draw a copy the Complex Rey's figure through immediate recall.<br>Ability to draw a copy of the Complex Rey's figure through delayed recall, after being administered a distracting task.
BVN BATTERY: TEST OF IMMEDIATE MEMORY DURING LEARNING OF A LIST OF WORDS[2,3]	BVNLi	Learning of a list of words according to the Buske-Fuld technique. The examinee is tested for his/her performance in the immediate recall.
BVN BATTERY: TEST OF DELAYED MEMORY DURING LEARNING OF A LIST OF WORDS[2,3]	BVNLd	Learning of a list of words according to the Buske-Fuld technique. The examinee is tested for his/her performance in the delayed recall.
BVN BATTERY: IMMEIDATE RECALL IN PROSODIC MEMORY[2,3]	BVNPi	Immediate recall of a short story.
BVN BATTERY: DELAYED RECALL IN PROSODIC MEMORY[2,3]	BVNPd	Delayed recall of a short story, after being administered a distracting task.
IMMEIDATE RECALL IN POSITIONAL MEMORY SUPRASPAN[2,3]	SUPRASPANi	Learning of visuo-spatial memory supra-span. The examinee is tested for his/her performance in the immediate recall.
DELAYED RECALL IN POSITIONAL MEMORY SUPRASPAN[2,3]	SUPRASPANd	Learning of visuo-spatial memory supra-span. The examinee is tested for his/her performance in the delayed recall, after being administered a distracting task.
TEMA TEST FOR LEARNING OF COUPLES OF WORDS[4]	TEMA	Learning of a list of couples of words. The test consists of two parts:<br>• couples of semantically related words<br>• couples of semantically unrelated words

Bibliography

1. Shin MS, Park SY, Park SR, Seol SH, Kwon JS (2006). Clinical and empirical applications of the Rey-Osterrieth Complex Figure Test. Nat Protoc 1 (2): 892–9.
2. Bisiacchi P, Cendron M, Gugliotta M, Tressoldi PE, Vio C. BVN 5-11 - Batteria di valutazione neuropsicologica per l'età evolutiva. (2009). Erickson, Trento.
3. Gugliotta M, Bisiacchi,PS, Cendron M, Tressoldi PE, Vio C. BVN 12-18 - Batteria di Valutazione Neuropsicologica per l'adolescenza. (2009). Erickson, Trento.
4. Reynolds CR, Bigler ED. (1994). Test of memory and Learning (TOMAL). Italian Edition: Test TEMA, a cura di (curated by) Ianes D. (1995). Erickson, Trento.

Supplementary Material 3

Trajectories of performance over the 20 sessions of treatment for 'Exp' group. The therapists were left free to select some (at least 3) out of 7 standard exercises at any session. They scored the performance corresponding to each exercise, session and patient.

Trajectories 1 to 10 refer to single patient. Trajectories from one patient are missing, as they could not be recorded by the therapist.

Exercise 1 (figure below). **The therapist asked and encouraged the patient to lift and stand up from the floor.**

Procedure: **The therapist encouraged the patient to move, from lying on a carpet on the ground, to all fours. If successful, the patient was asked to kneel and then to lift ond leg, maintaining on knee only on the mattress. Then, the patient was encouraged to securely stand up in upright position.**

This exercise was administered to 4 children in at least 1 session. The graph shows that participants n° 4, n° 5 and 10 performed well since the beginning of intensive treatment; consequently, the exercise was substituted with other requests. In this exercise, ceiling effect is observed also for patient n°1.

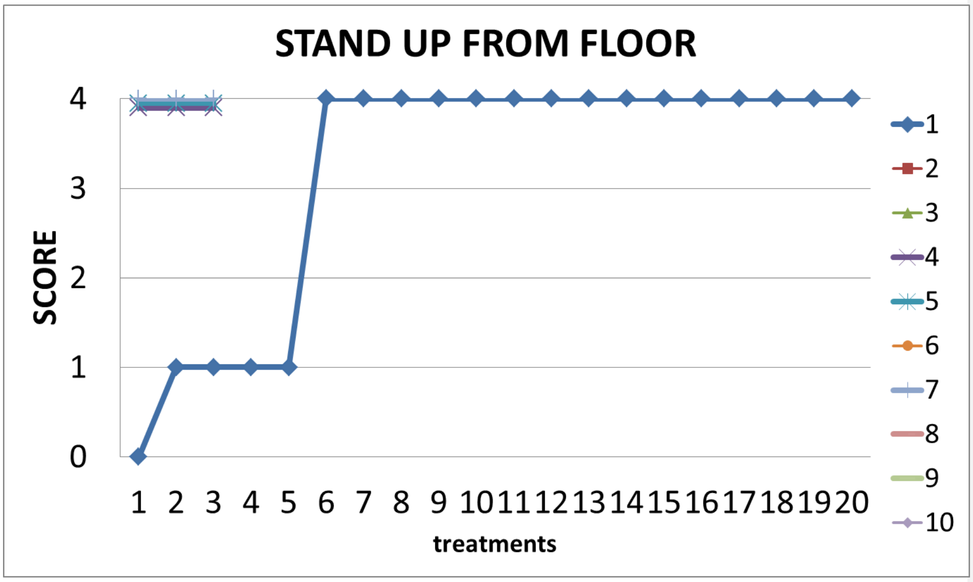

Score derives from the accomplishment of the following tasks:

1. **on all fours**
2. **kneel**
3. **one knee on the ground**
4. **standing**

Exercise 2. **The therapist asked and encouraged the patient to undress. This exercise was administered to 4 children in at least 1 session. The graph shows that participants n° 8 and 9 improved the performance rapidly, over 3-4 subsequent sessions.**

Procedure: **The patient was put to seat on a carpet on the floor. The therapist encouraged the patient to unlace and then take off their shoes, one at a time. Then the patient was asked to take off his/her socks and tuck them into the shoes. Then the therapist asked the patient to take out the pullover and to fold it for storage.**

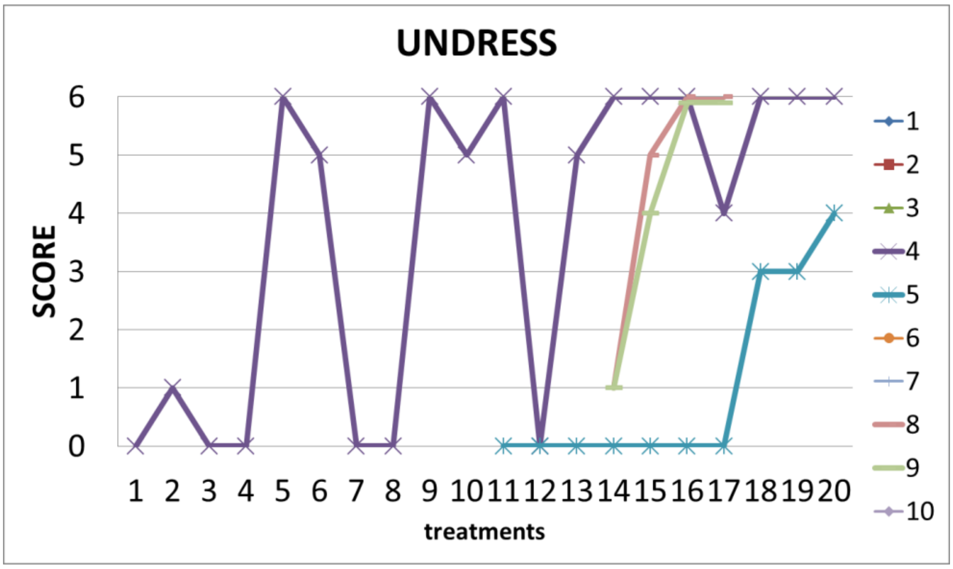

Score derives from the accomplishment of the following tasks:

1. **Unlace his own shoes**
2. **Take off his own shoes**
3. **Take off his own socks**
4. **Put the socks in his own shoes**
5. **Remove the sweatshirt**
6. **Fold the sweatshirt**

Exercise 3. **The therapist asked and encouraged the patient to build a pyramid with some blocks. This exercise was administered to all patients in at least 1 session. The graph shows that patients n°2 and n°6 scored on top since the beginning of the intensive treatment (first session); patients n° 6 and 9 improved the performance rapidly, over the first 6/8 sessions. Patients n°3, as well as others, gradually increased their performance, despite certain variance.**

Procedure: **The patient was put to seat on a carpet on the floor. The therapist encouraged the patient to stack 5 blocks vertically, from the largest (side = 10 cm) to the smallest (side = 3 cm).**

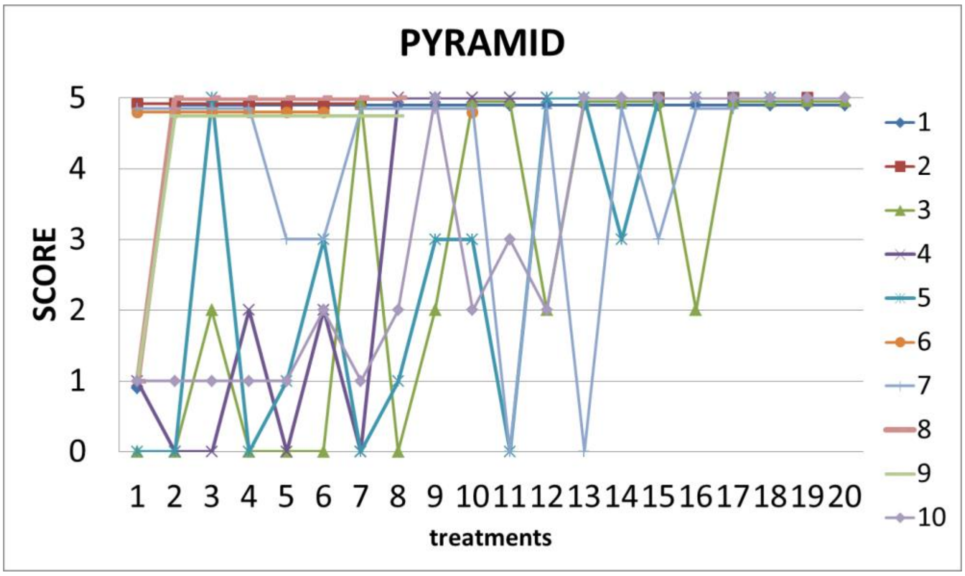

Score derives from the accomplishment of the following tasks:

1. **stack up 1 block**
2. **stack up 2 blocks**
3. **stack up 3 blocks**
4. **stack up 4 blocks**
5. **stack up 5 blocks**

Exercise 4. **The therapist asked and encouraged the patient to perform some manual tasks with tools (ergotherapy) in the room. This exercise was administered to all patients in at least 1 session. The graph shows a general trend of improvement in performance for all patients. Patient n°4 performed scored on top since the beginning. Ceiling effect is observed for patients n°3, n° 4 and n° 8.**

Procedure: **The therapist encouraged the patient to switch on the room light. When the patient was successful, the therapist then encouraged to turn a handle to open the room door. Then, the patient was supervised while opening the window through rotating a handle (15 cm long, rotating 90 degrees and after positioning the patient at the right height by means of a support), and last he/she was asked to open a zip or snap-fastener.**

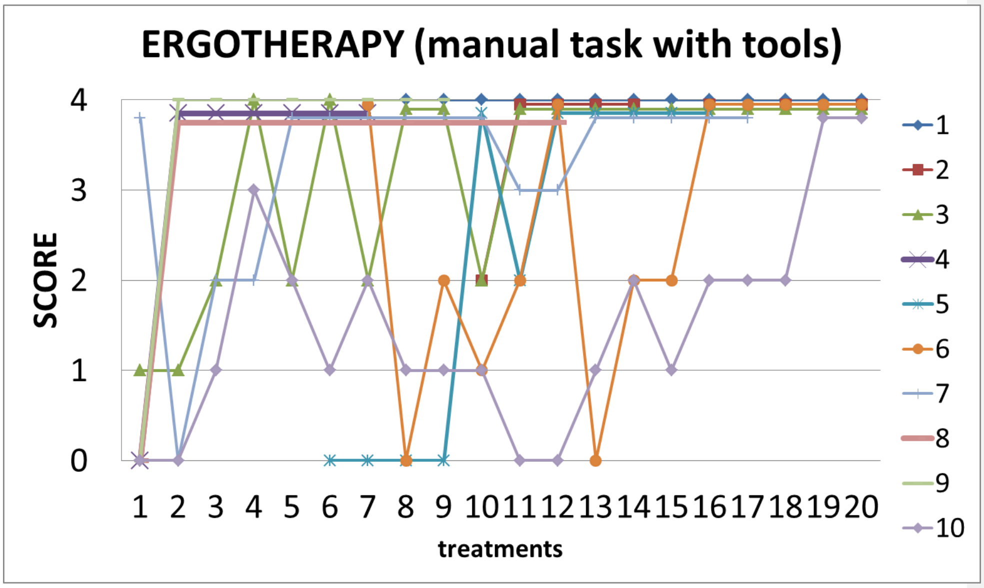

Score derives from the accomplishment of the following tasks:

1. **switch on the room light**
2. **turn the door handle**
3. **open a window**
4. **open a zipper or snap-fastener**

Exercise 5. **The therapist asked and encouraged the patient to perform a trail task. This exercise was administered to 8 patients in at least 1 session. The graph shows a general trend of improvement in performance for all patients. Ceiling effect is observed for patients n°1 and n°8.**

Procedure: **The therapist proposed the patient to walk within a path (with no walking aids but with therapist's assistance if and where needed). The path was built with cones, bars and hoops placed on the ground. If successful, the patient was asked to overcome an obstacle placed at the end of the path (15 cm of height from the ground), and then enter a hoop perpendicular to the ground (60 cm of diameter). Then, he/she was asked to pass another obstacle (20 cm from the ground), and last to slalom between cones and clubs.**

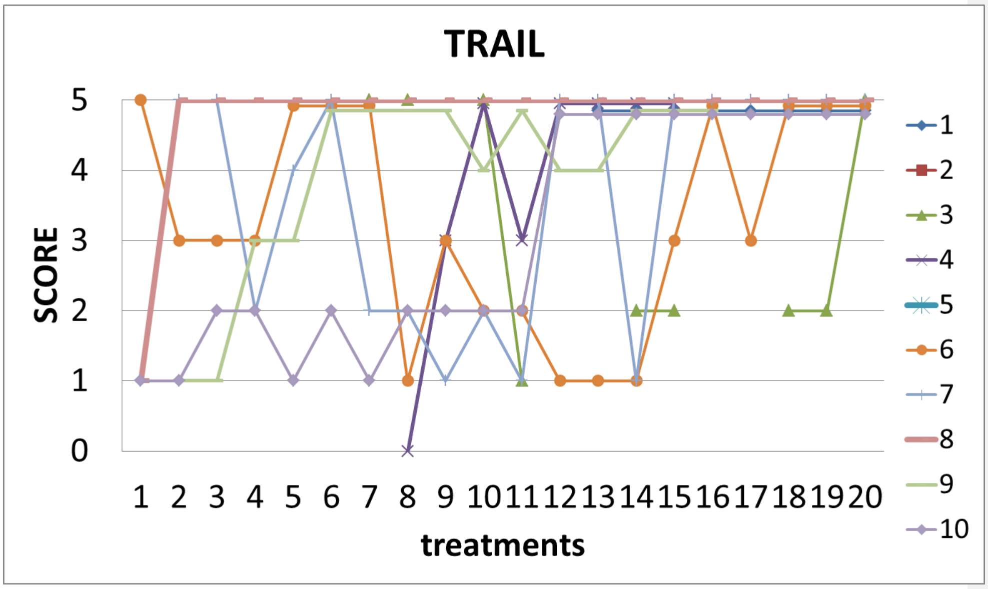

Score derives from the accomplishment of the following tasks:

1. **walk within a path**
2. **pass an obstacle**
3. **enter in a hoop**
4. **pass an obstacle (other type than in 2.)**
5. **slalom among cones/clubs.**

Exercise 6. **The therapist asked and encouraged the patient to prepare some coffee, by handling a coffee pot. This exercise was administered to 8 patients in at least 1 session. The graph shows that nearly all the patients improved the performance rapidly, over the first 6/8 sessions after the one in which the task was proposed for the first time. Patient n°6 scored on top of scale since the beginning; trajectory of patient n°2 shows relevant ceiling effect for this exercise.**

Procedure: **The therapist proposed the patient to prepare a pot of coffee. The patient was asked to open the coffee pot, and then to pour water inside the pot. Then, the patient was asked to insert, put in place and (try to) adjust the coffee filter. Then, he/she was asked to put some grounded coffee inside by using a tea spoon, and to close the coffee pot.**

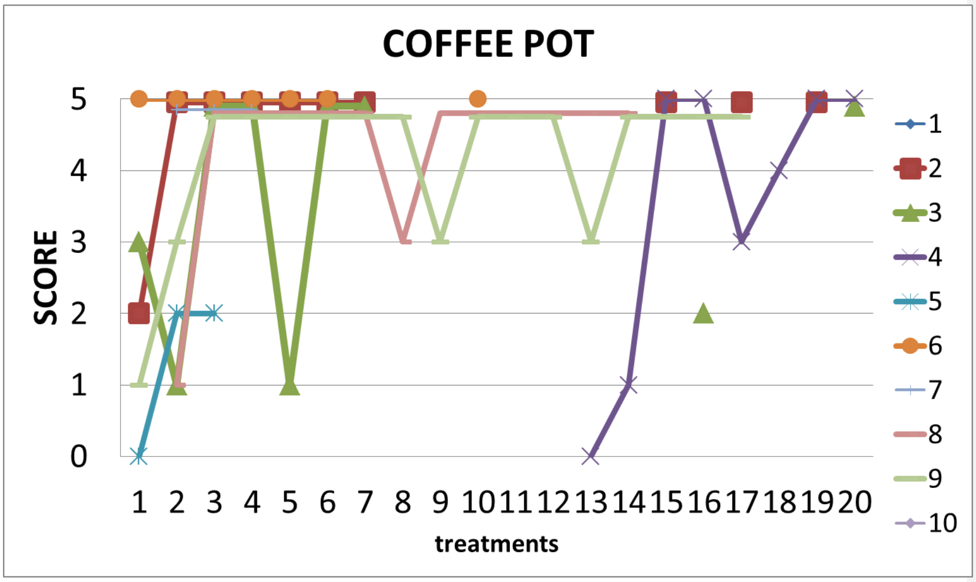

Score derives from the accomplishment of the following tasks:

1. **open the coffee pot**
2. **pour water inside**
3. **insert, put in place and (try to) adjust the coffee filter**
4. **add coffee**
5. **close the coffee pot**

Exercise 7. The therapist asked and encouraged the patient to wash his/her teeth. This exercise was administered to 6 patients in at least 1 session. The graph shows that participants n°3 and n° 5 performed well since the first time the exercise was proposed, thus, with ceiling effect for this exercise. In the case of patient n°3, the task was substituted after session 5. Other patients, such as n°4 and n°9, rapidly improved their performance, within 2/4 sessions.

Procedure: The patient was put standing in front of a basin, prepared for the occupational therapy. The toothpaste and personal toothbrush were placed beforehand in a glass container attached to the wall and at adjusted height. The therapist proposed the patient to open the cap of a toothpaste tube with both hands, grab their own toothbrush with one hand, squeeze the toothpaste on the toothbrush with the assisting hand, close the toothpaste tube with both hands and wash their own teeth with the toothbrush and water, by employing one hand to brush and the assisting hand to manage the tap water.

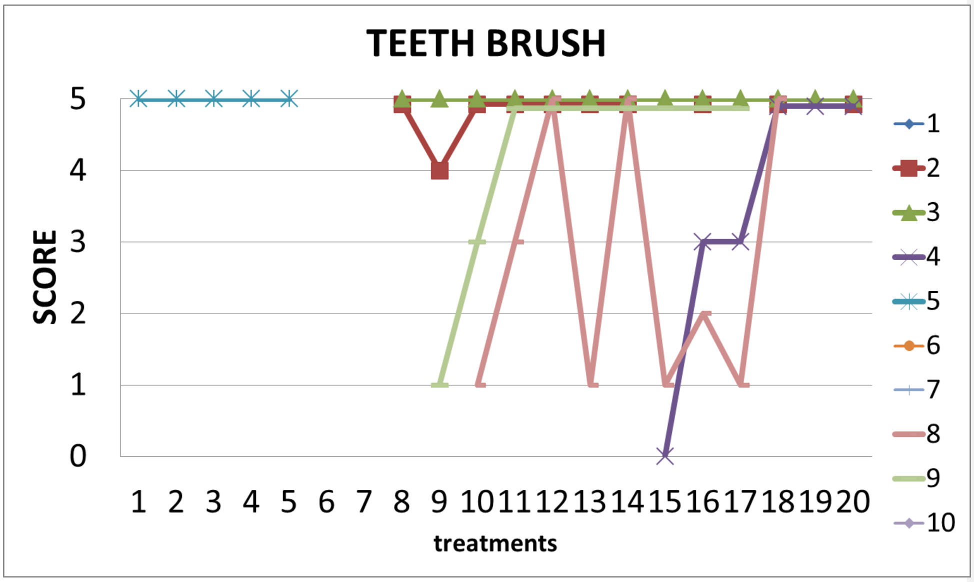

Score derives from the accomplishment of the following tasks:

1. **open the toothpaste tube**
2. **grab the toothbrush**
3. **squeeze the toothpaste on the toothbrush**
4. **close the toothpaste tube**
5. **wash his own teeth with the toothbrush and water**

Supplementary Material 4

4.1 MRI acquisition

Patients in the ‘Exp’ group underwent brain MRI before and after treatment. Participants in the ‘Hlt’ group underwent one MRI session only. MRI acquisition was performed using a 3.0 T imaging scanner (Philips Medical Systems, Best, the Netherlands). Resting state functional MRI (rsMRI) scans were acquired using a T2*-weighted single-shot echo-planar imaging sequence during rest (repetition time [TR]/echo time [TE]=2000/30 ms; flip angle=90°; field of view=240 mm^2; matrix size=96×96; slice thickness=4 mm; 250 sets of 30 contiguous axial sections). All patients were instructed not to move inside the scanner and were encouraged to keep calm and still by trained personnel. In addition, we acquired a three-dimensional (3D) T1-weighted Turbo-Field-Echo sequence (TR/TE=8/4 ms; flip angle=8°; 170 contiguous axial sections; matrix size=224×224; field of view=200 mm^2) and a T2-weighted Turbo-Spin-Echo sequence (TR/TE=3000/100 ms; flip angle=90°; 110 contiguous axial sections; matrix size=144×144; field of view=224 mm^2).

4.2 Functional MRI analysis

T1-w and T2-w images were corrected for field inhomogeneity by applying N4 Bias Field Correction algorithm (Tustison et al., 2010). rsMRI data were realigned with MCFLIRT (Jenkinson, Bannister, Brady, & Smith, 2002) in two steps: (1) rigid registration and (2) affine realignment. After applying BET algorithm to T2-w data for aiding registration (Smith, 2002), mean volume of rsMRI realigned sequences was registered to a common anatomical space by a three-step procedure (Nordio, Peruzzo, & Arrigoni, 2016): (1) a non-linear registration of the rsMRI mean volume to the skull-stripped T2-w data, (2) a rigid registration between T2-w and T1-w scans, and (3) a non-linear registration between T1-w and MNI standard space (Fonov et al., 2011). All registration steps were performed by employing the Advanced Normalization Tools (ANTS) (Avants, Tustison, & Song, 2009). For the

step (3) we computed masks of the lesions for each patients to exclude their influence on the normalization. Final resolution was set to 3x3x3 mm^3. Data were smoothed with a 6-mm Gaussian kernel.

rsMRI data were cleaned from artefactual and noise contributions by applying Independent Component Analysis (ICA), as implemented in FSL MELODIC software (Beckmann & Smith, 2004). For each subject, the number of components was calculated automatically by the software; single resulting components were visually inspected for final acceptance. For a component to be accepted, the corresponding ICA spatial map had to be coherent with known resting-state networks (RSNs), and had to have a spectral content highly concentrated in the low frequency range (< 0.1 Hz).

Group analysis was conducted by multi-session temporal concatenation, as implemented in MELODIC. The set of spatial maps from the group-average analysis was used to generate subject-specific versions of the spatial maps and associated time-series, using dual regression (Beckmann, Mackay, Filippini, & Smith, 2009). First, for each subject, the group-average set of spatial maps was regressed (as spatial regressors in multiple regression) into the subject's 4D space-time dataset. This resulted in a set of subject-specific time-series, one per group-level spatial map. Next, those time-series were regressed (as temporal regressors) into the same 4D dataset, resulting in a set of subject-specific spatial maps, one per group-level spatial map. To examine intra-network functional connectivity we tested for group differences using FSL's *randomise* permutation-testing tool (Filippini et al., 2009), applied on the resulting spatial maps. Basic network modelling of the time-series data were performed to study differences in partial correlation among the RSNs (inter-networks functional connectivity), using FSLNets script. For the intra-network functional connectivity analysis a threshold of $p < 0.05$ (corrected for multiple voxel comparison) was considered significant. Analogously, for the inter-networks functional connectivity analysis a threshold of $p < 0.05$ was considered (corrected for multiple networks comparison in this case).

References

Avants, B., Tustison, N., & Song, G. (2009). Advanced Normalization Tools (ANTS). *Insight Journal*, 1–35. Retrieved from ftp://ftp3.ie.freebsd.org/pub/sourceforge/a/project/ad/advants/Documentation/ants.pdf

Beckmann, C. F., & Smith, S. M. (2004). Probabilistic Independent Component Analysis for Functional Magnetic Resonance Imaging. *IEEE Transactions on Medical Imaging*, *23*(2), 137–152. https://doi.org/10.1109/TMI.2003.822821

Beckmann, Mackay, Filippini, & Smith. (2009). Group comparison of resting-state FMRI data using multi-subject ICA and dual regression. *NeuroImage*, *47*(Suppl 1), S148. https://doi.org/10.1073/pnas.0811879106

Filippini, N., MacIntosh, B. J., Hough, M. G., Goodwin, G. M., Frisoni, G. B., Smith, S. M., … Mackay, C. E. (2009). Distinct patterns of brain activity in young carriers of the APOE-epsilon4 allele. *Proceedings of the National Academy of Sciences of the United States of America*, *106*(17), 7209–14. https://doi.org/10.1073/pnas.0811879106

Fonov, V., Evans, A. C., Botteron, K., Almli, C. R., McKinstry, R. C., & Collins, D. L. (2011). Unbiased average age-appropriate atlases for pediatric studies. *NeuroImage*, *54*(1), 313–327. https://doi.org/10.1016/j.neuroimage.2010.07.033

Jenkinson, M., Bannister, P., Brady, M., & Smith, S. (2002). Improved optimization for the robust and accurate linear registration and motion correction of brain images. *NeuroImage*, *17*(2), 825–841. https://doi.org/10.1016/S1053-8119(02)91132-8

Nordio, A., Peruzzo, D., & Arrigoni, F. (2016). Use of T2-weighted 3D acquisition for correction of EPI-induced distortion in fMRI. In *ISMRM*. Singapore: ISMRM.

Smith, S. M. (2002). Fast robust automated brain extraction. *Human Brain Mapping*, *17*(3), 143–155. https://doi.org/10.1002/hbm.10062

Tustison, N. J., Avants, B. B., Cook, P. A., Zheng, Y., Egan, A., Yushkevich, P. A., & Gee, J. C. (2010). N4ITK: Improved N3 bias correction. *IEEE Transactions on Medical Imaging*, *29*(6), 1310–1320. https://doi.org/10.1109/TMI.2010.2046908

Supplementary Table 5

Power calculation for GROUP effect, GROUP variable being ne=11 'Exp' and nc=10 'Ctl' participants. Alpha (type 1 error) is set to 0.05. 'F-value' and 'Significance' are referred to ne+nc=21; 'Partial Eta Squared' is the effect size and 'Observed power' is (1-$\boldsymbol{\beta}$) with $\boldsymbol{\beta}$ being the type II error.

TEST	F value	Significance	Partial Eta Squared	Observed Power ($\alpha = 0.05$)
REY	0.004	0.952	<0.001	0.050
BVNLi	2.307	0.160	0.110	0.234
BVNLd	2.120	0.167	0.131	0.274
**BVNPi**	**8.894**	**0.015**	**0.497**	**0.756**
BVNPd	4.552	0.062	0.336	0.478
SUPRASPANi	0.601	0.456	0.057	0.108
SUPRASPANd	1.508	0.248	0.131	0.199
**TEMA**	**21.296**	**0.001**	**0.640**	**0.988**

In bold, we list the measures which provided significance in this study.
